# Brain targeting based nanocarriers loaded with resveratrol in Alzheimer's disease: A review

**DOI:** 10.1049/nbt2.12127

**Published:** 2023-03-22

**Authors:** Cyrus Jalili, Amir Kiani, Mohammadreza Gholami, Fariborz Bahrehmand, Sajad Fakhri, Somayeh Kakehbaraei, Seyran Kakebaraei

**Affiliations:** ^1^ Medical Biology Research Center Research Institute for Health Technology Kermanshah University of Medical Sciences Kermanshah Iran; ^2^ Regenerative Medicine Research Center Research Institute for Health Technology Kermanshah University of Medical Sciences Kermanshah Iran; ^3^ Department of Anatomical Sciences Kermanshah University of Medical Sciences Kermanshah Iran; ^4^ Pharmaceutical Sciences Research Center Health Institute Kermanshah University of Medical Sciences Kermanshah Iran; ^5^ Student Research Committee Tabriz University of Medical Sciences Tabriz Iran; ^6^ Faculty Anatomy Students Research Committee Kermanshah University of Medical Sciences Kermanshah Iran

**Keywords:** β‐amyloid, Alzheimer’s disease, Nanoparticles, Neuroinflammation, Resveratrol

## Abstract

Alzheimer's disease (AD) is one of the chief neurological difficulties in the aged population, identified through dementia, memory disturbance, and reduced cognitive abilities. *β*‐amyloid (Aβ) plaques aggregations, generation of reactive oxygen species, and mitochondrial dysfunction are among the major signs of AD. Regarding the urgent need for the development of novel treatments for neurodegenerative diseases, researchers have recently perused the function of natural phytobioactive combinations, such as resveratrol (RES), in vivo and in vitro (animal models of AD). Investigations have shown the neuroprotective action of RES. This compound can be encapsulated by several methods (e.g. polymeric nanoparticles (NPs), solid lipid nanoparticles, Micelles, and liposomes). This antioxidant compound, however, barely crosses the blood–brain barrier (BBB), thereby limiting its bioavailability and stability at the target sites in the brain. Thanks to nanotechnology, the efficiency of AD therapy can be improved by encapsulating the drugs in a NP with a controlled size (1–100 nm). This article addressed the use of RES, as a Phytobioactive compound, to decrease the oxidative stress. Encapsulation of this compound in the form of nanocarriers to treat neurological diseases to improve BBB crossing is also discussed.

## 1 INTRODUCTION

**FIGURE 1:**
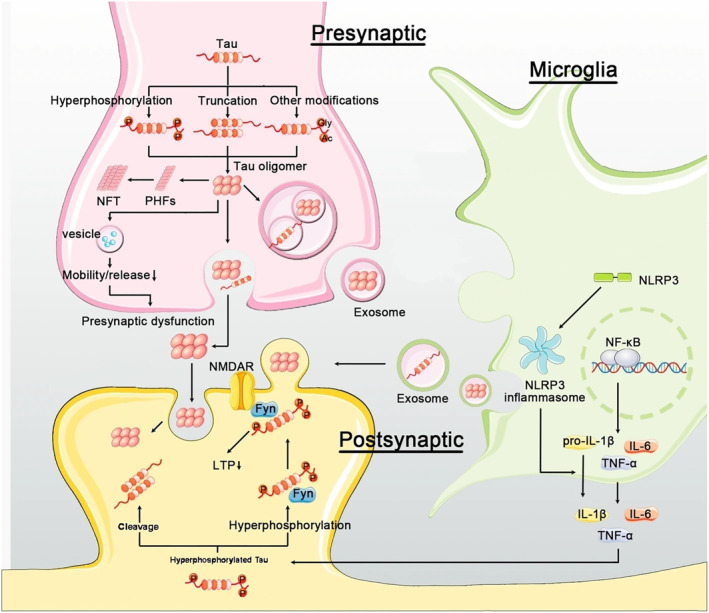
A model for tauopathy (Modified by [6]).

Alois Alzheimer found the presence of Aβ and neurofibrillary tangles (NFTs) in Alzheimer's patients [1]. By 2030, 14%–20% of people are estimated to live with Alzheimer's disease (AD). Neuroinflammation is the main cause of neurodegenerative disorders [2]. The prevalence of AD is expected to rise as the lifestyle in the world leads to the gradual disturbance of mental, cognition, and learning capabilities [3]. AD is known as a pathological progressive disturbance caused by cumulation, unusual sedimentation, and scattering of central nervous system (CNS) Aβ plaques, filamentous interneuronal NFTs, and then death of neurons and atrophy in the temporofrontal cortex [4]. In AD, the NFTs experience an unusual agglomeration of hyperphosphorylated Tau, which starts in the transentorhinal cortex and deploys throughout the cerebral stratum [5]. Moreover, Tau is a microtubule‐related protein capable of diverse post‐translational modifications (PTMs) (such as glycosylation, N‐terminal truncation, and phosphorylation). In the case of abnormal PTMs, the P‐Tau aggregation and its oligomer forms (NFTs and paired helical filaments) occur in the nervous system with the detachment of P‐Tau from microtubules. Finally, aggregated Tau forms are sent into the extracellular matrix via exosomes and vesicles [6]. Furthermore, Aβ and Tau are produced in the neuronal cells and can arrive at microglia via various mechanisms. Expression of the microglial cells by tauopathies can directly activate the NF‐κB inflammation pathways and NLR family pyrin domain containing 3 (NLRP3) protein, leading to an increase in pro‐inflammatory cytokine and its secretion [7, 8]. In tauopathies like AD, significant hyper phosphorylation and cleavage of Tau variants can enter postsynaptic spaces to destroy long‐term potentiation within the hippocampal segment by regulating the Src family tyrosine kinase Fyn/N‐methyl‐d‐aspartate (NMDA) receptor complex (see Figure 1) [9]. Yet, the primary reasons for the formation of amyloid‐β (Aβ) have not been elucidated in AD. Nevertheless, the hypothesis of the Aβ plaques proposes mutations or sequential cleavage in the Aβ precursor protein (APP) [10]. Moreover, an imbalance between Aβ production and its removal plays a fundamental role in the progression of AD [11]. Aβ plaques congregate in the blood vessels and brain tissue. The results revealed a direct correlation between imbalanced elimination and generation of reactive oxygen species (ROS) and Aβ‐induced cytotoxicity with AD progress [12]. Current strategies for treating AD and Parkinson's diseases (PD) can be categorised into five groups: anti‐inflammatory drugs, gene therapy, neuroprotective medicines, stem cells, and neurotrophic agents. Some of these treatment methods (e.g. stem cells) are very expensive. All the mentioned methods have side effects to some grade. Therefore, the development of the safe, efficient, and affordable drugs that can be prescribed for a long time is highly welcome [13]. However, the use of antioxidants has shown great promises in the treatment of nervous system disorders. The efficacy of resveratrol (RES) has been reported in neurodegenerative disorders, thereby, it may also be beneficial in AD [14]. Among the various pharmacological and therapeutic characteristics of RES as a stilbene flavonoid, the neuroprotective function of RES has received more attention. The RES can protect the neuron cells via antioxidant properties, anti‐amyloidogenic, anti‐tauopathy, anti‐inflammatory, Aβ plaques degradation, and preventing the mitochondrial DNA (mtDNA) damage [15, 16, 17]. Diverse mechanisms have been proposed for the neuroprotective behaviour of RES [18, 19]. Nevertheless, research works alone cannot clarify all the aspects of the RES effect on AD. In this regard, the commercial application of RES as a pharmaceutical drug is currently facing various limitations in vivo; especially, due to its low bioavailability, poor solubility, and quick metabolism [13, 20]. Recent progress in RES carrier delivery can help reduce the adverse effects of high doses of RES, not only in cancer therapy, but also in other diseases [21, 22]. Evidence has shown that the opposite results from in vivo studies of RES may be due to its low bioavailability [23]. However, poor bioavailability of RES can be resolved by the nanoencapsulation technique. Various types of drug carriers have been examined to boost the low bioavailability and stability of RES, thus, lowering the required RES doses and its corresponding side effects [24, 25]. This review emphasises novel advances in nanocarriers (NCs), for boosting the therapeutic efficacy of RES as a potential candidate for the prevention and treatment of neurological disorders like AD. In this regard, the current paper is aimed to overview the promising approaches that can be useful in the NC‐based delivery of RES to the brain tissue and its pharmacokinetics applications.

## 2 RESVERATROL

RES (3, 5, 4′‐trihydroxystilbene) is a natural polyphenol that can be found in grape skins, plums, peanuts, bilberries, and dark chocolate. RES is one of the most effective components of grape juice [26]. This phytoalexin was first elicited in the medical herbal white hellebore. Subsequently, it was detected in the polygonum cuspidatum, which was used in Chinese herbal medicine as an anti‐inflammatory and antifungal agent. RES can exist in geometric arrangements (cis/trans) isomerism from, being trans‐form the most therapeutic applications [27]. It can be changed into the cis‐form upon exposure to UV radiation (ultraviolet) [28]. Experiments have indicated that RES is very well endured by experimental models with no serious effects. The orally administered RES in rats and dogs (with respective doses of 200 and 600 mg/kg/day) for 90 days led to no obvious side effects [29]. Besides, the RES dosage of 450 mg/day is safe for a 60‐kg normal person [30]. Despite the numerous human and animal studies that emphasised the effective and protective properties of RES [31, 32, 33], limited number of clinical works have addressed the adverse effects of RES.

However, the high efficacy in biologically positive effects of RES elevates the level of antioxidant enzymes, including superoxide dismutase (SOD) activity, catalase (CAT), and glutathione peroxidase (GPx), and reduces ROS [34]. Moreover, it has been reported that RES has different therapeutic efficacies, like modulation of many cell‐signalling pathways [35], antioxidant, anti‐ageing attributes [36], anti‐inflammatory, anticancer [37], and neuroprotective (such as AD and PD) [38]. Studies have shown that RES as a polyphenolic stilbene supplement is involved in various pathophysiological functions of AD [39, 40]. According to the chemical and physical properties and the mechanism of its interaction with extracellular and intracellular molecules, RES has anti‐amyloidogenic properties and can be promising against neuroinflammation and inhibition of AD [41].

### 2.1 Biological properties of resveratrol

#### 2.1.1 Antioxidative activities

The enhancement of ROS in the biological system plays a decisive role in AD as it reacts with the fundamental biological molecules, interferes with the oxidant‐antioxidant balance in cells and organs, injures cells, and leads to cell death [42]. Moreover, the signalling action of ROS in AD influences mtDNA mutations, oxidative damage, Aβ metal ion redox reactions, and cessation of DNA protein cross‐links [43]. The favourable results of RES can be assigned to its antioxidant and free radical scavenging properties [44, 45]. In other studies, RES reduced the ROS levels in the H19‐7 cell line against Aβ through restrained peroxidation of membrane lipids and decreased the oxidative stress‐induced adverse effects [46]. Oxidative damage stimulates *β* and *γ*‐secretes (amyloid precursors), which explains the accumulation and the genesis of Aβ [47]. Based on Wang et al., RES diminished ROS cumulation by enhancing mitochondrial biogenesis through a complex PGC‐1alpha (peroxisome proliferator‐activated receptor *γ* coactivator 1α) and Sirt1 (Sirtuin 1) pathway [48]. Studies proved that promoting ROS is associated with the cognitive dysfunction perceived in AD [49, 50]. Moreover, RES could improve cognitive dysfunction by enhancing the CAT activity and GPx in the brain tissue and reducing serum levels of malondialdehyde and nitrite/nitrate in the AD rats model [51]. In addition, it can suppress abnormal increases in reactions of oxidative lipids in vivo and effectively scavenge intracellular ROS [52]. Zhang et al. showed the ability of RES to protect dopaminergic neurons against lipopolysaccharide (LPS) neurotoxicity by prohibiting the expression pathway of NF‐κB (the nuclear factor kappa B)‐mediated neuroinflammatory respone [53]. An increase in lipid peroxidation is a prevalent risk factor for AD that reduces membrane integrity and full permeability to selective ions like calcium in the plasma membrane, confirming the connection between NF‐κB and particular fragments of DNA, which leads to cell death and tissue injuries [54, 55]. According to Chen et al., RES reduced the mitochondrial injury and apoptosis induced by neuroexcitotoxic stress in the hippocampal CA3 region of LPS‐treated mice [56]. RES directly enhances the Nrf2 (the nuclear factor erythroid‐2‐related factor 2) that stimulates the mRNA gene expression templates of antioxidant enzymes against glutamate‐induced LPS of HT22 cells, playing a remarkable role in the supporting cells [57]. Studies have also assigned the promising antioxidative function of RES in an in vivo model of AD to heme oxygenase‐1 expression and activity [58]. RES, as a bioactive compound, could counteract the harmful impacts of oxidative damage by activating the expression of p38–MAPK (mitogen‐activated protein kinase) and preventing the expression of pathway cyclooxygenase, nitric oxide (NO) synthase [59], tumor necrosis factor‐α (TNF‐α), and interleukins (IL‐1β, IL‐6, IL‐12, IL‐23) [60]. Additionally, results have shown that RES inhibits oxidative agents by (I) decreasing reaction nitrogen species and ROS production, (II) free radical scavengers, (III) enhancing antioxidant defense‐related gene expression (e.g. Nrf2, glutathione, and CAT), and (IV) the control of mitochondrial biogenesis, mainly by activating Sirt1 and Nrf2, extracellular signal‐regulated kinase/p38 mitogen‐activated protein kinases (MAPK) signalling pathways [61].

#### 2.1.2 Anti‐inflammatory activities

**FIGURE 2:**
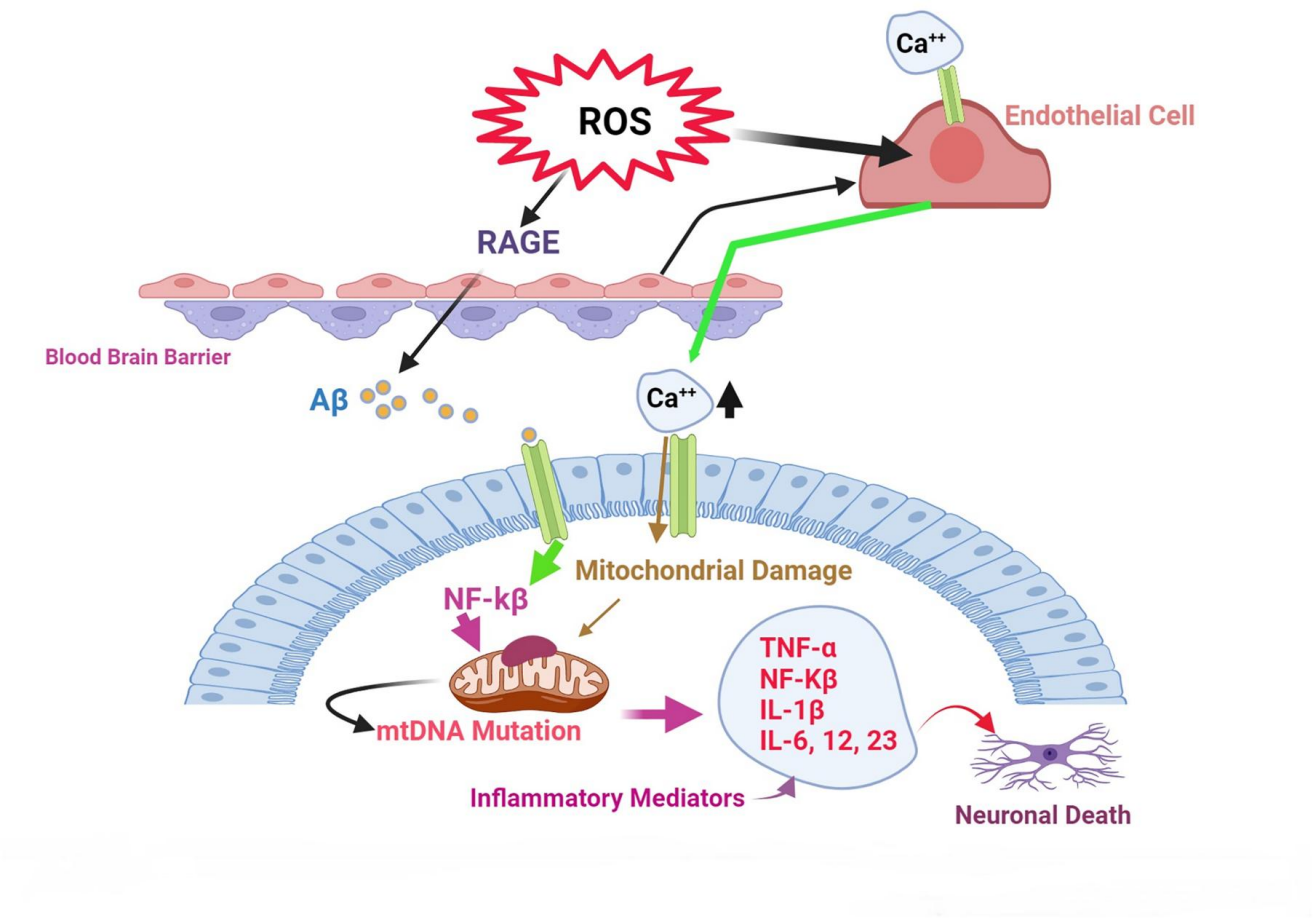
Increased reactive oxygen species (ROS) is a risk factor for Aβ accumulation and Alzheimer's disease development.

High levels of prolonged free radical injury increases inflammation within the brain. An enhancement in oxidative damage biomarkers can also disturb the homoeostatic balance in inflammatory cytokines, inducing the death of nerve cells [62]. A recent study proposed that people with lower antioxidant capacity are rather vulnerable to oxidative stress [63]. The accumulation of senile plaque‐containing intraneuronal Aβ peptides in AD causes microglial activation‐induced acute neuroinflammation, which leads to neuronal degeneration and eventually enhanced necroptotic cell death in hippocampal neuron cells [64]. Another proposed mechanism of RES relies on its ability to restrain the proinflammatory signalling molecules related to AD. Feng and Zhang reported that RES considerably deterred the proliferation of Aβ‐induced microglial activation BV‐2 cells with reduced generation of proinflammation factors IL‐6, iNOS, IL‐1β, and TNF‐α [65]. The neuroprotective and anti‐neuroinflammatory abilities of RES are via inhibition of NF‐κB. Zhao et al. reported that RES exerted neuroprotection and anti‐apoptotic properties in vivo against Aβ1–42 induced CNS inflammation, potentially prevented by the NF‐κB signalling pathway‐dependent mechanism [66]. Moreover, RES inhibits prostaglandins (PGE2), NO, TNFα, cyclooxygenase‐1 (COX‐1), and NF‐κB activity in LPS‐exposed rat primary microglia cultures [67, 68]. Interestingly, RES can decrease receptor for advanced glycation end products (RAGE) protein expression (receptor for advanced glycation products) in the hippocampus region and suppress the expression of matrix metalloproteinase‐9 (MMP‐9), which has been found accountable for junction proteins [66]. RAGEs at the blood‐brain barrier (BBB) are the main gate for Aβ peptide transportation to the brain [69]. In addition, RES inhibits the action of Quinone reductase 2 at a low micro molar concentration by providing persistence against ROS‐related neuronal damage (see Figure 2) [70, 71]. Dasgupta et al. reported that RES is an activator of AMP‐activated protein kinase (AMPK) and reduces the Aβ levels and deposition, a potent regulator of the survival of neuronal cell line exposed to oxidative damage [72]. Moreover, RES support mitochondria against LPS by a combination of arachidonic acid and iron through a mechanism that involves AMPK‐dependent phosphorylation of glycogen synthase kinase‐3beta (GSK3β) [73]. Another study focussed on treatment with RES‐supported SK‐N‐BE cells against Aβ aggregation‐induced cell toxicity via an enhancement in Sirt1 expression that restrained the hydrogen peroxide (H_2_O_2_) [74] and Aβ generation in AD by suppressing the *α*‐secretase gene ADAM10. The regulation of the Notch signalling is a principal pathway in cell‐fate [75]. One of the molecular pathways of RES‐mediated neuroprotection involves the expression of the Sirt1 gene, which can retain neuron cells against oxidation, inflammation, apoptosis, and necrosis [76]. These findings support the idea that the Sirt1 gene might achieve remedial objectives for neurodegenerative disorders, for instance, AD and PD [77]. In vitro results proved that RES remarkably prevented Aβ25–35 induced neurotoxicity of primary mouse cortical neurons and can enhance expression of Sirt1 to preserve neurons' survival against oxidative stress [78]. The protective effect of this bioactive compound directly prevents the cyclic adenosine monophosphate phosphodiesterase's signalling pathway and leads to the activation of the molecular mechanism of AMPK and Sirt1 [79]. Data confirm that Sirt1 overexpression leads to neuroprotective in AD [80], because Sirt1 prevents the NF‐κB signalling pathway by reducing Aβ‐induced neurotoxicity in primary cortical cell culture [81]. The bioactivity of RES not only plays a significant role in ROS and NO scavenging but can also activate AMPK independently of Sirt1 [71], neuroinflammation prevention, enhancing glutathione levels, diminution of proinflammatory factors (i.e. TNF‐α, IL‐β, COX, iNOS) [46]. Furthermore, it has been established that RES can activate the N‐terminal region of Sirt1 [82], decreasing Aβ of accumulation, protecting against neurotoxicity, and apoptosis [83]. Neuroinflammation has a crucial impress in the induction of AD [84]. Findings have indicated the presence of inflammatory biomarkers in the adult brain with AD, for example, enhancing the proinflammatory cytokines/chemokines in cerebrospinal fluid (CSF) and serum, along with symptoms of micro gliosis [85]. So, the enhancement in the level of these inflammatory markers is related to cognitive disorders at various phases of AD directly.

#### 2.1.3 Anti‐amyloidogenic activities

**FIGURE 3:**
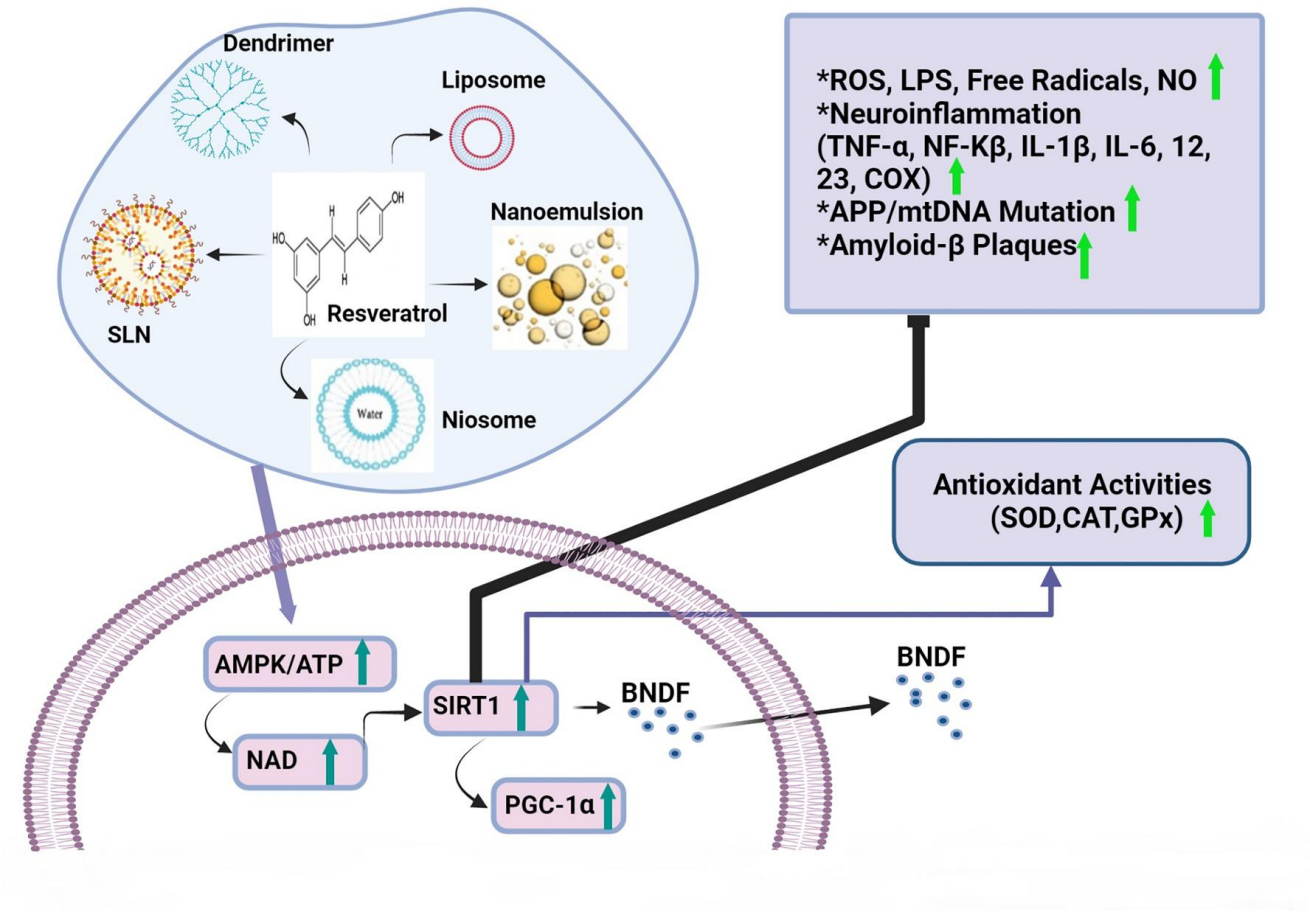
Resveratrol (RES) antioxidant effects via scavenging reactive oxygen species (ROS), activation of Sirt1/AMPK, and notable efficacy in improving memory.

AD mainly occurs through specific pathological‐based factors such as Aβ deposition and accumulation of hyper‐phosphorylation of P‐Tau with the constitution of NFTs [86]. The aggregation of Aβ molecules can be assigned to the generation of Aβ plaques on the nerve cells in the brain, resulting in neuronal damage in AD patients [87]. Aβ monomers, particularly Aβ1−42, quickly form self‐aggregates to produce Aβ oligomeric forms with synaptotoxic effects which can also reduce the density of the dendritic spines throughout the CA1 hippocampal layers [88]. RES is a natural compound that intervenes with the Aβ accumulation and inhibits the construction of the supposed toxic Aβ oligomers, offering a proper candidate for the prevention and remedy of AD [14, 89]. RES inhibits Aβ fibrils (Aβ1−42) and Alzheimer's neuritic plaques by connecting to the N‐terminus region of Aβ monomers [90]. Additionally, RES inhibits the formation of Aβ plaques by connecting to the transthyretin transporter protein [91] via the activation of Sirt1, regulation of the *α*‐secretase [92], and ubiquitin‐like protein, with a significant role in proteostasis [93]. RES also prevents memory loss in AD by diminishing high levels of complexes IV mitochondrial (or cytochrome c oxidase) and proinflammatory (IL‐6, IL‐1β, and TNF‐α) in the rat brain neurons via the activation of Sirt1 and AMPK pathways [94, 95]. A recent work has determined that the anti‐amyloidogenic function of RES decreased the secretion of Aβ to the extracellular space and Aβ accumulation by activating CaMKKβ pathway‐dependent phosphorylation of AMPK‐activating kinases [96]. Moreover, NFTs are constructed of abnormal aggregation of P‐Tau and plaques made up of Aβ 1–42, which cause progressive nerve cell death in the brain [97]. The P‐Tau injuries affect glial action, which leads to adverse outcomes for glial cells, astrocytes, and non‐cell autonomous results on the safety and structure of neurons [98]. Schweiger et al. represented that RES as an antioxidant compound diminishes phosphorylation of P‐Tau and causes improved behaviour and cognitive disorder in vitro and in vivo [99]. It has been reported that RES can inhibit tauopathy by reducing P‐Tau in a mouse AD model [51]. The potential capability of RES stimulates the dephosphorylation of the microtubule‐dependent P‐Tau via the inhibition of GSK‐3β and calcium/calmodulin‐dependent protein kinase II (CaMKII) [100], and the gene overexpression of Sirt1 induces the deacetylation of acetylated Tau [101]. The investigations offer that RES could boost cognitive action in the senile, induce neuroplasticity, and enhance brain‐derived growth factor via activation of the Sirt1/AMPK pathway in the adult brain [39, 48]. The results of Sirt1 upregulation by RES can prevent neuronal cell death by deacetylating p53, NF‐κB, and Forkhead box O, there preventing Aβ accumulation and neurodegeneration in AD [102, 103]. Other findings illustrated that the protective actions of RES via overexpression of Sirt1, metal chelation, and ROS scavenging diminished the malondialdehyde and nitrite levels, preventing the formation of P‐Tau and Aβ and the induction of AD [104, 105]. The RES put anti‐inflammatory effects against Aβ‐related neuronal damage and cell death by enhancing the activity of Sirt1, inhibiting the pathway of the HMGB1/TLR4/MyD88/NF‐kB and the inflammatory cytokines/chemokine in the brain [106]. Porquet et al. represented that RES decrease the inflammatory reaction in the AD mice model by reducing ROS production and asserted that exists a level of balance concerning Sirt1/AMPK signaling associated with inflammatory changes, which is needed for the RES protective functions against Aβ aggregation and cognitive plaque damage (see Figure 3) [94]. Both in vitro and in vivo results prove that RES can be a safe treatment for AD. Noteworthy, although the currently available supplements and medications can temporarily decrease the symptoms of AD, they cannot easily pass the BBB and stop the progress of brain damage [83]. The BBB hinders the transmission of pharmaceutical agents, posing a serious challenge for all the macromolecule drugs, which fail to cross the BBB. Almost 98% of small molecules cannot freely pass through the BBB [107]. On the other hand, the BBB is a dynamic construction that includes micro vessel endothelial cells, astrocytes, and perivascular cells that prevent the crossing of biological medicines into the cellular microenvironment and blood flow of the brain and permit the passing of nutrients. Therefore, novel strategies for the favored improvement of neurological disturbance depend on medicine bioavailability [108]. Evidence shows that the metabolism of RES mostly happens in the liver, although it has poor stability and bioavailability, its metabolites pass through the BBB [109] in a small amount and can be easily detected in the CSF and plasma of people [110]. The efficiency of medicine forwarding through the BBB significantly belongs to the features such as hydrophilicity, differentiation degree, molecular size etc. The capability of NCs to pass via the BBB pertains to their physical and chemical specifications [111]. In nanoscience, the Nano encapsulation technique is to carry drugs throughout the organs and provide a protective coating around diverse bioactive implicate. The absorption, pharmacokinetic factors, and bioavailability of RES can be upgraded to treat neurological diseases [112]. Several studies show that creating nanomaterial‐based encapsulation for RES is advantageous in passing through the BBB and brain tissue [113, 114]. The exact mechanisms of RES are unknown, but as a therapeutic agent, there seems to apply impressive effects in vitro and in vivo.

## 3 NANOCARRIERS

### 3.1 Nanocarriers as an emerging platform for drug delivery to the blood–brain barrier

The BBB has significant transmission mechanisms for the porter of nutritional and solute molecules into the brain. Drugs may cross the BBB via receptor‐mediated transcytosis. Research efforts have been focused on the development of novel methods based on in‐deliver drug‐conjugated nanoparticles (NPs) [115], liposomes [116], albumin NPs, and solid lipid nanoparticles (SLNs) to the CNS [117, 118]. Small pharmaceutical drugs may be designed to cross the BBB by endogenous transport systems within the cerebral capillary endothelium. In addition, macromolecules (recombinant proteins, peptides, and antisense oligonucleotide radiopharmaceuticals) can pass across the BBB via molecular Trojan horse technology [119]. Lipid NCs have revolutionized clinical and pharmaceutical applications by pharmacology expansion of various drugs, allowing them to pass the BBB without disrupting the intercellular junctional complexes [120]. In addition, lipid‐based NCs are a landmark in drug delivery with several promising physicochemical features including biosafety, long circulation time in blood flow, ability to cross different barriers, cellular uptake, tiny size, specific pharmacokinetic characteristics, capability to adsorb different molecules on their surface, the feasibility of incorporating hydrophilic and hydrophobic chemicals, and high stability [121, 122]. Thanks to their high specificity and sensitivity, NCs can offer a desirable platform for the transfer of drugs across the BBB. It is necessary to mention that NCs‐mediated drug transportation to the brain tissue highly depends on the type of surfactant. A study reported the use of 12 various surfactants onto the surface of poly‐butyl cyanoacrylate NCs [123]. Accordingly, the NCs with polysorbate 20, 40, 60, and 80 coatings could successfully promote the transfer of several medicines across the BBB [124]. NCs can permeate the brain through diverse strategies including passive transport, carrier‐mediated transport, receptor‐ and absorptive‐mediated transcytosis, and para cellular transport. Non‐invasive techniques that interrupt BBB totality also facilitate the transfer of NP across the BBB [125]. The probable mechanism for the transmission of drugs across the BBB relies on the phospholipid bilayer of SLN; moreover, liposome facilitates the infiltration of drugs across diverse biological membranes. However, it does not permit crossing BBB [126, 127], thus, different surface modifications have been made to enable the delivery of these NCs via BBB.

### 3.2 The role of various types of nanocarriers as a therapeutic tool for Alzheimer's disease

The convolution of the therapeutic approach to AD lies not only in its obscure aetiology and the absence of the present effective therapies, but also in the limited access to the affected organ. In addition to limitations caused by the BBB in the transfer of drugs, other factors, such as clearance mechanisms remarkably degrade the half‐life of the drug in the body, thus, reducing its effectiveness [128]. The use of NCs also faces various limitations, especially in the field of medicine which can be listed as follows: (I) the effect of temperature on the solubility of NPs, which can influence the NP‐drug interaction [129], (II) the administration method of the NPs and its efficacy on the bioavailability of drugs [130], (III) the BBB functions as an impermeable barrier to a large number of exogenous substances such as drugs [131], (IV) toxicity, and (V the costly production of NPs which requires specific equipment, and optimal conditions, especially for NPs that can be used for therapeutic targets [132]. These challenges can be overcome by the use of NCs for drug transfer due to their high surface‐to‐volume ratio and the possibility of the effective functionalisation of NCs with targeting ligands to further promote their passage across the BBB [133]. The combination of NP_S_ with zinc, lactoferrin‐conjugated N‐trimethylated chitosan, and polysaccharides has revealed non‐toxicity, high biodegradability, stability, and hydrophilicity [134, 135, 136]. The polymeric poly‐ethylene‐glycol (PEG)‐formulated NPs improved the treatment of memory problems and significantly decreased the Aβ peptides, neuronal damage, neuroinflammation, and cognitive impairment [137]. Studies showed that the composition of Nano micellar with Tween‐80 and PEG ceramide enhanced the effectiveness of nano‐micelles in destroying Tau protein and the regulating mechanism of autophagy in target cells [138, 139]. Another method to improve the efficacy and effectiveness of the drug for CNS disorders is dendrimer compounds with ethylene diamine which enhance the solubility and bioavailability of the medicine, further improving the permeation over BBB to target injured areas of the brain [140]. The dendrimers‐based NCs is found the most efficient strategy in the targeted drug delivery to inhibit the formation of amyloid deposits [141]. At the moment, chitosan and tripolyphosphate nano‐gels are the best drug carriers capable of loading macromolecules, active molecules, and drugs with great potentials in the treatment of various types of CNS diseases such as AD [142]. Solid lipid NPs can be employed as excellent carriers for both lipophilic and hydrophilic drugs, which have shown remarkable inhibitory effects against aggregation of Aβ. Nowadays, nanostructured drug‐loaded lipid carriers and solid lipid NPs have displayed neuroprotective effects against hallmarks of Aβ and neurochemical variations in AD with higher bioavailability and effectiveness in the brain [143, 144]. The biggest advantage of using these NPs is as promising tools in the theranostics for a vast range of Combined Therapies, like magnetic resonance imaging (MRI) of the body, drug delivery together of phototherapy, chemo/phototherapy of cancer, and positron emission tomography imaging‐guided [145]. Liposomes as safe Nano medicine can aid the prosperous forward of targeted drug delivery across the brain tissue by using diverse polyether's, functional proteins, and cell‐penetrating peptides [146]. In addition, the liposomes as potential carrier systems can notably raise the transfer of medicines to CNS through corresponding receptors on BBB cells and the protective efficacies for hippocampus neurons and anti‐Aβ attributes in AD therapy [147]. Niosomesas as NC systems are vesicles combined with non‐ionic groups surfactants, which are biodegradable, non‐toxic, have high stability and solubility, that can improve brain functions in CNS disorders [148]. Thus, this formulation with combined pentamide‐loaded chitosan and glutamate‐coated niosomes can be used as a potential approach to overcome Aβ neurotoxicity and AD therapy [149]. Both in vivo and in vitro experiments reveal the promising effects of Nano emulsion preparations against amyloid genesis and AD pathologies [150]. On the other hand, cubosomes are another smart lipid‐based NP with a wide range of pharmaceutical uses, such as delivering drugs to the brain‐afflicted parts [151]. Cerium, selenium, iron, and gold show significant anti‐AD properties, and scientists are interested in synthesising metal NPs. ROS inhibitors include selenium (II), sodium selenite (VI), and sodium selenite (IV) [152]. Substantial micronutrients in the human body (selenium Nano formulations, selenium, and selenite NPs), which are used in biomedicine widely, play a crucial role in reducing oxidative damage and treating neurological disorders such as AD [153]. Cerium oxide NPs could effectively scavenge free radicals, reduce oxidative damage, and preserve the neuronal cells against high ROS levels in an AD patient [154]. The various Gold NP formulations are played a fundamental role in delivering medicine through the BBB to the brain, which can promise strategies in the therapy of AD. Results from another research show that treatment with gold NPs in mouse models of AD has remarkably moderated the signs of AD by decreasing neuroinflammation, improving spatial learning and memory, and reducing Oxidative injury‐related mitochondrial disarrangements [155]. The iron oxide NPs formulations can act as inhibitors of Tau aggregation in rat primary cortical neurons to cure AD. Also, biomedical sciences indicate iron oxide NPs may contain an application potential to ameliorate early detection and treatment of neurodegenerative illnesses like AD [156].

## 4 RESVERATROL‐LOADED NANOPARTICLE IN ALZHEIMER'S DISEASE

The development of new treatment methods to meliorate nervous system disorders, can be promoted by using new drug delivery technologies (NPs or Bioactive nanomaterials) [157]. Recently, the US Food and Drug Administration has approved several drug‐encapsulating NCs as the most useful approach for treating neurodegenerative diseases, including AD. These systems facilitate the drug transfer to the damaged parts of the brain (see Table 1). Nanotechnology plays a pivotal role in these strategies to improve and enhance the durability, solubility, penetration, transfer the bioactive compounds to various organs including the brain [158]. Lipid NCs have exhibited excellence features compared to other NCs (e.g. liposomes, polymeric, Nano emulsions, and nanoniosomes) due to their capacity to dominate BBB, as lipid NPs can simulate low‐density lipoprotein particles [159, 160]. The infusion of RES‐nano capsules has protected brain activity by increasing the intracerebral condensation of RES in mouse. It can also enhance the solidity of the molecules of RES in the brain and substantially prohibit synaptic impairment [161]. Solid lipid nanoparticles consist of solid lipids (triglycerides) which can organise the transport of colloidal RES to the brain when tween 80 surfactant was used in their structure [162, 163]. Moreover, studies have reported the ability of NPs to protect bioactive compounds from degeneration, enhancing the topical transfer of RES and increasing its oral bioavailability [164, 165]. Neves et al. evaluated the effects of RES‐SLNs with apolipoprotein E through a transwell method using the hCMEC/D3 cells. The results indicated the ability of RES‐SLNs to pass the BBB, where SLNs remarkably enhanced the permanence of RES (1.8‐fold better) through the BBB compared to free‐RES [162]. The ameliorated performance of RES‐loaded nano‐capsules can be attributed to the capability of NCs to increase the content of RES in the brain. In vitro experimental model of rat hippocampal demonstrated that NCs meliorated the neuroprotection effects of RES against oxidative stress‐induced Aβ and decreased the secretion of inflammatory agents (TNF‐a, IL‐1b, IL‐6, IL‐10) which improved learning problems and suppressed the c‐Jun N‐terminal kinases activity pathway [166]. Similarly, the RES‐loaded SLNs improved in‐memory efficacy and cognitive ability by decreasing ROS in dementia and AD through enabling the Nrf2/OH1 signalling pathway and inhibiting the aggregation of Aβ [14, 167]. In the animal model of AD, encapsulation of RES‐Se (RES‐selenium) NPs by SLN was successful in the treatment of memory disorder by upregulating the expression of Sirt1 along with PI3K protein and downregulating STAT3 (Signal transducer and transcription activator) expression as well as decrementing the IL‐1β level [168]. Therefore, NC systems seem to possess remarkable potential in alleviating neuroinflammation and treating AD [169]. Polymeric nanoparticles (PNPs) based on biodegradable copolymers (from synthetic or natural sources) can be used for the transportation of the drugs in the treatment of AD. In experiments on the transgenic mice *Caenorhabditis elegans*, RES‐PNPs decreased Aβ‐induced neurotoxicity by reducing free radical scavengers and over‐regulation of SOD‐3 expression [170]. Thanks to their high biocompatibility and tunable hydrophilic and hydrophobic surface, micelles (amphiphilic molecules) have found numerous biological applications including the development of smart pharmaceutical carriers to target some zones of the body (diameter of 10–100 nm). A study shows that RES‐encapsulated neuronal mitochondria‐targeted micelle (CT‐NM/RES) can remarkably enhance the condensation RES in the neuronal mitochondria and promote compatibility between mitochondrial split and merger, which restituted cognitive function in APP/PS1 transgenic AD model mice [171]. In another experiment, RES‐loaded polymeric micellae NPs diminish caspase‐3 activity and ROS against the Aβ‐induced oxidative stress in PC12 cells [169]. Lately, researchers have concentrated on compounding RES into cross‐linked chitosan microspheres/NPs with vanillin for ameliorating the stabilisation and increasing the encapsulation performance of RES in microspheres by 93.68% [172]. However, SLNs can be employed as a carrier for RES as they enhance the durability, solubility, and intracellular transfer of RES [173]. SLN has excellent encapsulation efficiency of about 94%. On the other hand, SLN remained stable for 3 months. Not only does SLN ameliorate the anti‐aggregation effect of RES, but it also conveys RES through the BBB model and prohibits the formation of Aβ‐plaques in the brain (see Table 2) [14]. However, the nasal (olfactory region)‐to‐brain route is another promising strategy to facilitate the transmission of the drug through the BBB to reach the CNS tissue [174]. When the drug enters the nasal cavity through the vestibular portion, its biomolecules are further moved to the olfactory regions transported to the CNS through various pathways mediated by vascular, trigeminal nerve, and olfactory nerve [175, 176]. Recent studies on animal models (AD and PD) reported the encapsulation of RES in cobosome, lipid carrier, nanoemulsion, nanosuspension, and polymeric nanoparticles. The intranasal drug delivery route can increase the bioavailability and permeability, protect neurons of brain tissue, ameliorate behavioral impairments, and cause molecular changes (see Table 2). Despite the excellent advantages of intranasal administration of the drugs to enter the CNS, (including self‐administration, affordability, rapid absorption, and non‐aggressive), some disadvantages as toxicity, rapid clearance, chemical degradation of the drug, and the low liquid volume for the dissolution of drug, may restrict this route [174, 177]. Emerging proof displays that suppression of neuroinflammation via NC‐mediated delivery of antioxidant drugs to overactivation microglial inhibition can be promising therapy for degenerative nerve disorders [178]. Microglia are the tissue‐resident immune cells of the brain accounting for 5%–12% of cells in the CNS. Microglia play a substantial role in preserving the neural circuits, responding to damage, mediating neuronal transition, and regulating synaptic and apoptosis [179, 180]. Specifically, microglial overactivation is an early reaction to the developing pathological change in the CNS, which leads to oxidative damage and the generation of a broad spectrum of pro‐inflammatory cytokines (IL‐1β, IL‐6, TNF‐α, and NF‐κB), and activation of tauopathy‐induced inflammasome NLRP3 pathway [181]. The primary outcomes of the studies illustrate that administration of RES‐loaded RAW‐Exo (exosomes derived from macrophages) via nasal route inhibited pro‐inflammatory factors, which can potentially target microglia in the brain and peripheral nervous system of a mouse model of multiple sclerosis, in vivo [182]. For example, epigallocatechin gallate (EGCG) and RES have been demonstrated to inhibit the Tau aggregation and cellular oxidative damage while exhibiting quench free radical‐producing enzyme expression (including nicotinamide adenine dinucleotide phosphate oxidases), in vitro, which is responsible for the activation of microglial and the overproduction of ROS‐induced neuronal apoptotic death [183, 184, 185]. Previous research has shown that silver NPs, polymeric NPs, poly‐e‐caprolactone, PEG‐based NPs, ceria‐zirconia, and mesenchymal stem cell (MSC)‐derived exosome reduced brain damage and pro‐inflammatory cytokine production in vitro via inhibiting microglial activation [178, 186, 187, 188]. From a therapeutic perspective, microglia can be easily targeted with various types of NPs due to their intrinsic phagocytic nature as macrophage cells of the brain for the treatment of diseases such as AD [189, 190]. Understanding the structure and function of microglia can assist in the design of suitable nanomedicines to pass the BBB and reach the brain. NPs interact with the microglial cell membrane and are internalised mainly through active transport (endocytosis), which comforts the uptake of the extracellular vesicles via biological membrane invagination [191]. Currently, due to the lack of recognition of molecular and cellular pathways, there is no decisive cure for AD. However, the use of antioxidants to improve symptoms is available and research is ongoing to find a suitable treatment. It is necessary to discover new strategies, AD biomarkers, and the production of advanced nanomedicines based on the use of antioxidants (RES as a neuroprotective) that have the least side effects that can be effective in targeting these biomarkers and treating this disease.

**TABLE 1:** FDA‐ Approved Nano medicines delivering for AD.

Carrier	Drug	Carrier material	Reference
Polymeric nanoparticles	Donepezil	Chitosan, PLGA (polysorbate 80‐coated), PLGA‐b‐PEG	Bhavna et al., 2014 & Baysal I et al., 2017 [192, 193]
	Galantamine	Chitosan, PLGA	Fornaguera C et al., 2015 [194]
	Rivastigmine HCl	Chitosan	Fazil M et al., 2012 [195]
	Rivastigmine Tartrate	PLGA, chitosan, PBCA	Joshi SA et al., 2010 & Khemariya RP et al., 2016 [196, 197]
Solid lipid nanoparticles	Galantamine	Glycerylbehnate (Compritol)	Misra S et al., 2016 [198]
	Lipoyl–Memantine	Stearic acid	Laserra S et al., 2015 [199]
	Rivastigmine HCl	Compritol 888 ATO	Shah B et al., 2015 [200]
Liposomes	Rivastigmine HC	Phosphatidylcholine; Dihexadecyl phosphate; cholesterol; glycerol	Ismail MF et al., 2013 [201]
	Donepezil	Carboxymethyl cellulose, 1,2‐distearyl‐sn‐glycero‐3‐phosphocholine, cholesterol, PEG	Al Asmari AK et al., 2016 [202]
CPP‐modified liposomes	Rivastigmine HCl	EPC, cholesterol, DSPE‐PEG‐CPP	Yang ZZ et al., 2013 [203]
Flexible liposomes	Galantamine	Soya phosphatidylcholine, cholesterol, and propylene glycol as edge activator	Li W et al., 2012 [204]

Abbreviations: CPP, Cell‐Penetrating Peptide; DSPE, 1,2‐Distearoyl‐*sn*‐Glycero‐3‐Phosphoethanolamine, EPC, Egg Phosphatidylcholine; PEG, Polyethylene Plycol, PBCA, Oolybutyl Cyano Acrylate; PLGA, poly Lactide‐co‐Glycolic Acid.

**TABLE 2:** NCs compounds and their production methods for RES delivery for AD therapy and intranasal delivery of RES.

Carrier	Material	Coating	Development phase	Major findings	Reference
SLN	Cetyl palmitate (solid lipid) & polysorbate 80 (surfactant)	ApoE	In vitro	RES‐ SLNs transfer via the BBB, where SLNs remarkably enhanced RES permanence (1.8‐fold better) in the BBB contrasted to free‐RES.	Neves et al., 2016 [162]
Lipid‐core nanocapsules	Poly (‐caprolactone), capric/caprylic triglyceride, sorbitan monostearate, polysorbate 80	No	In vivo	Improved bio diffusion of RES in the brain and decreased the harmful effects of Aβ on memory and learning, and reduced inflammatory factors.	Frozza et al., 2013 [166]
SLN	Solid lipid cetyl palmitate, polysorbate 80	DSPE‐PEG, LissRhod‐PE	In vitro	Enhancement of the anti‐aggregation function of RES, and the prohibition of amyloid plaques fibrillation.	Loureiro et al., 2017 [14]
SLN	Stearic acid, lecithin, Taurocholate	No	In vivo	Increase of the RES bioavailability, overexpression of Nrf2/HO1, and a decrease in degenerative change.	Yadav et al., 2018 [167]
Se‐NPs	Na2SeO3,Milli‐Q water	No	In vivo	Enhancing expression of Sirt1, PI3K protein, and reducing IL‐1β level, improvement in the neurocognitive ability.	Abozaid et al., 2022 [168]
PNPs	Methoxy‐polyethyleneglycol, caprolactone, acetone	No	In vivo, in vitro	RES alleviates damage from *γ* ‐ray radiation and Aβ ‐peptide neurotoxicity in *C. elegans* via ROS scavenging.	Yin et al., 2014 [170]
Polymeric micelles	Poly‐caprolactone, PEG	No	In vitro	RES protected PC12 cells from Aβ‐induced through decreasing oxidative and activity of caspase‐3 in a dose‐dependent manner.	Lu et al., 2009 [169]
CT‐NM	PEG‐PLA micelles, MPEG‐PLA, TPP‐PEG‐PLA micelles	No	In vivo	RES scavenged mitochondrial ROS effectively to decrease oxidative damage, decreased Aβ aggregation, and up‐regulated Sirt1 expression.	Yang et al., 2020 [171]
Nanostructured lipid carrier	Cetyl palmitate, Capmul MCM, acrysol, poloxamer 188, tween 80	In situ hydrogel (gellan gum, xanthan gum)	Intranasal delivery (in vivo)	Enhanced delivery of RES to the brain through the nasal mucosa	Rajput et al., 2018 [205]
Nanoemulsion	Labrasol, Transcutol, tween 80	Vitamin E	Intranasal delivery (in vivo)	Better bioavailability of RES in the brain, effective targeting ability of nanoemulsion when given intranasally	Pangeni et al., 2014 [206]
Polymeric nanoparticles	Polysorbate 80, dichloromethane, polyvinyl alcohol, and polylactide	N/A	Intranasal delivery (in vivo)	The neuroprotective action of RES‐loaded polymeric nanoparticles displayed against behavioural, cognitive, and chronic neurological changes induced by MPTP (1‐methyl‐4‐phenyl‐1,2,3,6‐tetrahydropyridine)	Lindner et al., 2015 [207]
Lipidic nanoemulsion	Labrafac lipophile, labarafac PG, Cremophor RH, tween 80	Hyaluronic acid	Intranasal delivery (in vivo)	Intranasal delivery indicated enhanced bioavailability of RES in the brain tissue	Nasr et al., 2016 [208]
Nanosuspension	Deacetylated gellan gum, ethanol	In situ gel (deacetylated gellan gum)	Intranasal delivery (in vivo	Bioavailability of RES in the brain tissue showed more than 2 twice increased availability in the intranasal route than in the intravenous route	Hao et al., 2016 [114]
Cubosomes	Glycerol monooleate, poloxamer 407	In situ gel	Intranasal delivery (in vivo	Res‐loaded cubosomes illustrated further permeability and elevated bioavailability in the brain in the intranasal route.	Ahirrao and Shrotriya, 2017 [209]

Abbreviations: AD, Alzheimer's disease; CT‐NM, neuronal mitochondria‐targeted micelle encapsulating; DSPE, 1,2‐Distearoyl‐sn‐Glycero‐3‐Phosphoethanolamine; MPEG‐PLA, Methoxy poly‐ethylene‐glycol‐poly‐lactide‐acid; NCs, nanocarriers; RES, resveratrol.

## 5 CONCLUSION AND FUTURE PROSPECT

RES shows several biological characteristics and medicinal effects, including anti‐tumor, anti‐inflammatory, anti‐tauopathy, Aβ plaques degradation, and anti‐oxidant actions. RES has insignificant bioavailability and extremely low oral absorption efficiency, restricting its clinical use. Nevertheless, clinical trials have not yet succeeded to display these actions, maybe due to the low bioavailability of RES, among other pharmacokinetics specifications. Therefore, studies have considered on the development and progress of RES‐Derivatives, which would preserve the neuroprotective attributes of RES, but can present higher efficacy. A suitable drug for the treatment of neurological diseases should enter the brain across the BBB. Regarding its low bioavailability and solubility, RES has a limited permeation across the BBB. Therefore, the bioavailability and stability of RES can be boosted by NCs and encapsulation methods. The NP‐drug delivery systems can protect the hydrophobic RES against enzymes, light, and pH. Such systems can also ameliorate drug‐targeting specificity in the CNS, enhance medicine loading efficiency, and improve the bioavailability. Studies on animal models of AD displayed successful delivery of the RES‐loaded NCs, but little information can be found on the mechanism of carrying drugs into or throughout the body.

Drug delivery methods can revolutionise the application of the bioactive compounds, as the development of novel molecules and the method of their transfer across the BBB could greatly affect the world. Furthermore, the investigations should be concentrated on the co‐delivery and target delivery of phytobioactive compounds to prohibit ROS that leads to neurological disorders such as AD. In this study, the anti‐amyloidogenic, anti‐inflammatory, and antioxidant activities of RES were described. Yet, the mechanisms of its function are not fully understood. Nanotechnology can contribute to the transport of RES to target site. The physico‐chemical features of the NPs, as well as their conditioned release and bioavailability, affects the phytobioactive formulations. Furthermore, most NPs have enhanced encapsulation efficiency, excellent durability, and extended release profile. These RES‐containing NCs have shown effectiveness in vitro and in vivo. The progress of NC‐based drug delivery techniques is more significant for transporting drugs to target organs by passing across the BBB. No biological agents have been recommended for the global clinical trials market so far. Their route of administration, along with in vivo instability, low penetration across the BBB, and high fabrication cost are major hurdles preventing the basic studies on the efficacy of potent pharmaceutical compounds. Furthermore, nanotechnology‐based drug delivery systems (synthetic polymer particles, SLN, liposome formulations, and micelles) can address these challenges and elevate the hopes for the treatment of AD by advancing biomedical research from animal models to clinical trials. In addition, these systems can offer promising features such as high bioavailability, targeting, imaging, stem cell, and gene therapy which can minimise off‐target side effects to further raise the treatment results. Valuable data on these potential strategies such as drug therapeutic and diagnostic research will be obtained from animal studies and other research works which are time‐consuming and require high research resources. With the growing global trend to seek for more accurate treatment and diagnosis, the smart and multi‐axial approaches based on NCs and nano‐drug delivery technology seem to have a bright future. As RES mainly targets Tau proteins, neuroinflammation, and Aβ plaques, there is an urgent need to develop approaches for its easier transfer across the BBB to enhance its efficiency and bioavailability. Novel strategies such as NCs that may not only cure symptoms but also prevent the development of neurological disorders at an early stage can improve the quality of life of the patients. Moreover, we conclude that in the future, emerging technologies in NCs can improve the capacity and potential ability of Res with regard to its antioxidant, neuroprotective function, and low adverse effects by establishing high bioavailability, permeability, and efficacy in the transmission of the BBB and management of the neurological disorders.

## AUTHOR CONTRIBUTIONS

**Seyran Kakebaraei**: Project administration, conceptualisation, supervision, visualisation, validation, writing ‐ review & editing, and data curation. **Cyrus Jalili**: Supervision, visualisation, and validation. **Amir Kiani**: visualisation and review editing. **Mohammadreza Gholami**: Visualisation and review editing. **Fariborz Bahrehmand**: Visualisation, review editing, and validation. **Sajad Fakhri**: Visualisation, review editing, and Validation. **Somayeh Kakehbaraei**; Visualisation, formal analysis, and Resources.

## CONFLICT OF INTEREST STATEMENT

The authors express that they have no conflict of interest in this study.

## Data Availability

Data sharing is not applicable to this article as no datasets were generated or analysed during the current study.
